# Ambulatory Follow-Up Visits After Emergency Department Discharge Among Medicaid Beneficiaries

**DOI:** 10.1001/jamanetworkopen.2024.41182

**Published:** 2024-10-25

**Authors:** Michelle P. Lin, Canada Parrish, Laura G. Burke, Ryan C. Burke, Amber Sabbatini

**Affiliations:** 1 Department of Emergency Medicine, Stanford University, Palo Alto, California; 2 Department of Emergency Medicine, University of Washington, Seattle; 3 Department of Emergency Medicine, Beth Israel Deaconess Medical Center, Boston, Massachusetts; 4 Department of Emergency Medicine, Harvard Medical School, Boston, Massachusetts; 5 Department of Health Policy and Management, Harvard T.H. Chan School of Public Health, Boston, Massachusetts

## Abstract

This cohort study examines Medicaid beneficiary visits to emergency departments (EDs) in Washington state from 2009 to 2017 to investigate the association between time to follow-up visit and 30-day ED revisits.

## Introduction

Medicaid beneficiaries frequently visit emergency departments (EDs) and also revisit EDs.^1,2^ Timely follow-up care after ED discharge may be associated with improved outcomes.^3,4,5^ Our objectives were to quantify follow-up visit rates after ED discharge among Medicaid beneficiaries and to test the association between time to follow-up visit and 30-day ED revisits.

## Methods

We examined ED treat-and-release visits among adult Medicaid beneficiaries (18-64 years old) in Washington state from 2009 to 2017. We generated a Kaplan-Meier curve to estimate the time-dependent probability of a follow-up visit after ED discharge. Follow-up visits were identified using *Current Procedural Terminology* codes 99201 to 99215. We fitted a Cox regression model with ED revisit as the outcome, time to follow-up as the exposure of interest, and 30 days as the censoring time and censored those experiencing an ED revisit or hospitalization before follow-up. We adjusted for hospital-level clustering using robust SEs and stratified by hospital to allow for unique baseline hazards. We adjusted for age, sex, race, ethnicity, chronic conditions, ED discharge diagnoses, and ED visits in the preceding 12 months. We examined subgroups with chronic conditions, including those with 3 or more nonpsychiatric chronic conditions.^6^ Consent was waived by the University of Washington Institutional Review Board because of the use of deidentified data. This cohort study was exempt from institutional review board review and adhered to the STROBE reporting guideline. The statistical analysis was performed from May 4, 2022, to December 8, 2023, using Stata software, version 16.1 (StataCorp LLC). Significance was defined as a 95% CI excluding 1.

## Results

We identified 2 680 765 total ED visits among 812 099 Medicaid beneficiaries (Table 1). Among these beneficiaries, 63.2% (n = 1 691 848) were female, the mean (SD) age was 37.6 (14.0) years, and 64.5% (n = 1 722 892) were White. Within 30 days, 37.3% (n = 1 000 017) completed follow-up visits, 24.8% (n = 663 513) completed an ED revisit, 3.1% (n = 83 322) were hospitalized, and 6.4% (n = 173 788) were censored because of an ED revisit or hospitalization preceding follow-up. Time to follow-up visit was associated with a 1.15% lower risk of a 30-day ED revisit (hazard ratio [HR], 0.985; 95% CI, 0.984-0.986) (Table 2), which was similar among those with 3 or more chronic conditions (HR, 0.980; 95% CI, 0.979-0.981) and 3 or more nonpsychiatric conditions (HR, 0.981; 95% CI, 0.980-0.983).

**Table 1.** Patient Characteristics Associated With Ambulatory Follow-Up Visits After ED Discharge

Characteristic	Overall (N = 2 680 765)	No follow-up within 30 d (n = 1 680 748)	Follow-up within 30 d (n = 1 000 017)
Age, mean (SD), y	37.6 (14.0)	36.3 (14.0)	39.7 (13.9)
Sex, No. (%)			
Female	1 691 848 (63.2)	1 016 085 (60.5)	675 763 (67.6)
Male	986 481 (36.8)	662 454 (39.4)	324 027 (32.4)
Race, No. (%)^a^			
American Indian or Alaska Native	148 966 (5.6)	101 635 (6.1)	47 331 (4.7)
Asian or Pacific Islander	99 074 (3.7)	60 467 (3.6)	38 607(3.9)
Black, non-Hispanic	269 239 (10.1)	177 018 (10.6)	92 221 (9.2)
White, non-Hispanic	1 722 892 (64.5)	1 064 265 (63.6)	658 627 (66.0)
Other	288 561(10.8)	181 645 (10.8)	106 916 (10.7)
Not provided	152 033 (5.7)	95 718 (5.7)	56 315 (5.6)
Hispanic ethnicity, No. (%)	354 575 (15.2)	223 869 (15.3)	130 706(15.0)
Comorbidities, No. (%)^b^			
Cancer	66 922 (2.5)	27 875 (1.7)	39 047 (3.9)
Cardiovascular	766 068 (28.6)	404 165 (24.1)	361 903 (36.2)
Cerebral	39 933 (1.5)	20 511 (1.2)	19 422 (1.9)
Central nervous system	324 897 (12.1)	166 983 (9.9)	157 914 (15.8)
Type 1 or 2 diabetes	325 412 (12.1)	161 228 (9.6)	164 184 (16.4)
Eye	80 978 (3.0)	34 768(2.1)	46 210 (4.6)
Genital	177 911 (6.6)	93 674 (5.6)	84 237 (8.4)
Gastrointestinal	571 379 (21.3)	294 710 (17.5)	276 669 (27.7)
Hematological	88 361 (3.3)	45 335 (2.7)	43 026 (4.3)
Infectious	217 617 (8.1)	123 254 (7.3)	94 363 (9.4)
Metabolic	259 042 (9.6)	138 804 (8.3)	120 238 (12.0)
Psychiatric	973 235 (36.3)	551 234 (32.8)	422 001 (42.2)
Pregnancy	390 874 (14.6)	255 858 (15.2)	135 016 (13.5)
Pulmonary	640 656 (23.9)	354 099 (21.1)	286 557 (28.7)
Renal	197 745 (7.4)	96 085 (5.7)	101 660 (10.2)
Skeletal	581 338 (21.7)	296 823 (17.7)	284 515 (28.5)
Skin	451 170 (16.8)	279 677 (16.6)	171 493 (17.2)
Substance use disorder	600 565 (22.4)	393 113 (23.4)	207 452 (20.7)
ED visits in prior year, mean (SD), No.	2.5 (4.1)	2.6 (4.3)	2.4 (3.8)

Abbreviation: ED, emergency department.

^a^ Racial categories, including other, are based on beneficiary self-report and provided in this format designated by the Washington Health Care Authority.

^b^ Comorbidity flags are based on the Chronic Illness and Disability Payment System, a diagnostic classification system that Medicaid programs can use to make health-based capitated payments.

**Table 2.** Adjusted Association Between Ambulatory Follow-Up Visits and 30-Day Return ED Visits Among Medicaid Beneficiaries After ED Discharge^a^

Characteristic	Hazard ratio (95% CI)
Completed ambulatory follow-up visit	0.985 (0.984-0.986)
Age	0.996 (0.995-0.997)
Female sex	0.958 (0.943-0.974)
Race (reference category, White)	
American Indian or Alaska Native	1.029 (1.012-1.045)
Asian or Pacific Islander	0.855 (0.833-0.877)
Black	0.978 (0.960-0.997)
Other	0.945 (0.913-0.977)
Hispanic ethnicity	0.922 (0.881-0.964)
Comorbid chronic conditions	
Cancer	1.122 (1.092-1.152)
Cardiovascular	1.064 (1.052-1.077)
Cerebral	1.009 (0.953-1.069)
Central nervous system	1.076 (1.069-1.085)
Type 1 or 2 diabetes	1.042 (1.033-1.051)
Eye	0.957 (0.924-0.992)
Genital	1.134 (1.112-1.157)
Gastrointestinal	1.091 (1.082-1.100)
Hematological	1.090 (1.023-1.160)
Infectious	1.022 (0.982-1.064)
Metabolic	1.091 (1.068-1.114)
Psychiatric	1.139 (1.127-1.151)
Pregnancy	1.109 (1.064-1.156)
Pulmonary	1.076 (1.067-1.085)
Renal	1.021 (0.968-1.077)
Skeletal	1.077 (1.064-1.089)
Skin	1.133 (1.098-1.169)
Substance use disorder	1.169 (1.154-1.184)
No. of ED visits in prior year	1.062 (1.055-1.068)

Abbreviation: ED, emergency department.

^a^ Adjusted for top 30 most common ED discharge diagnosis clinical classification software categories.

## Discussion

A shorter time to follow-up visit was associated with a lower HR of 30-day ED revisit; however, a minority of Medicaid beneficiaries (37.3%) completed a follow-up visit within 30 days. This modest yet consistent effect size suggests follow-up visits alone may not reduce ED revisits and that more intensive interventions (eg, to mitigate adverse social risk) are needed. Key limitations are generalizability outside of Washington state; administrative data without clinical severity or social risk, which may increase follow-up and ED revisits; and censoring due to frequent ED revisits. Our finding that more than half of Medicaid beneficiaries did not complete follow-up visits within 30 days of ED discharge suggests substantial potential to improve access to care and reduce ED revisits.

## Notes

Supplement.
Data Sharing Statement
