# Supplementary material for: Ambulatory Follow-Up Visits After Emergency Department Discharge Among Medicaid Beneficiaries
Source: JAMA Netw Open. 2024 Oct 25;7(10):e2441182. doi: 10.1001/jamanetworkopen.2024.41182 (PMC11581479; doi:10.1001/jamanetworkopen.2024.41182)
Supplement: Supplement. — Data Sharing Statement [file jamanetwopen-e2441182-s001.pdf]

Data Sharing Statement

Lin. Ambulatory Follow-Up Visits After Emergency Department Discharge Among Medicaid Beneficiaries. *JAMA Netw Open*. Published October 25, 2024.
doi:10.1001/jamanetworkopen.2024.41182

Data
Data available: No

Additional Information
Explanation for why data not available: Data sharing is not allowed per the terms of the Data Use Agreement executed with the Washington State Health Care Authority.
